# Predictors of Hospital Readmission within 30 Days after Coronary Artery Bypass Grafting: Data Analysis of 2,272 Brazilian Patients

**DOI:** 10.21470/1678-9741-2020-0266

**Published:** 2020

**Authors:** Camilla do Rosário Nicolino Chiorino, Vinicius Batista Santos, Juliana de Lima Lopes, Camila Takao Lopes

**Affiliations:** 1 Educação Corporativa da Associação Beneficência Portuguesa de São Paulo, São Paulo, Brazil.; 2 Programa de Pós-Graduação em Enfermagem, Escola Paulista de Enfermagem, Universidade Federal de São Paulo, São Paulo, Brazil.; 3 Departamento de Enfermagem Clínica e Cirúrgica, Escola Paulista de Enfermagem, Universidade Federal de São Paulo, São Paulo, Brazil.

**Keywords:** Myocardial Revascularization, Risk Factors, Patient Readmission, Postoperative Period

## Abstract

**Introduction:**

In order to reduce readmission rates after coronary artery bypass grafting (CABG), its predictors should be known in different contexts. The objective of this study was to identify predictive factors of hospital readmission within 30 days after CABG in a Brazilian center.

**Methods:**

A secondary analysis of an electronic database of patients submitted to isolated CABG was performed. The relationship between readmission within 30 days and demographic, anthropometric, clinical, and surgery-related characteristics was investigated by univariate analyses. Predictors were identified by multiple logistic regression.

**Results:**

Data from 2,272 patients were included, with an incidence of readmission of 8.6%. The predictors of readmission were brown skin color (Beta=1.613; 95% confidence interval [CI] 1.047-2.458; *P*=0.030), African-American ethnicity (Beta=0.136; 95% CI 0.019-0.988; *P*=0.049), chronic kidney disease (Beta=2.214; 95% CI 1.269-3.865; *P*=0.005), postoperative use of blood products (Beta=1.515; 95% CI 1.101-2.086; *P*=0.011), chronic obstructive pulmonary disease (Beta=2.095; 95% CI 1.284-3.419; *P*=0.003), and use of acetylsalicylic acid (Beta=1.418; 95% CI 1.000-2.011; *P*=0.05). Preoperative antibiotic prophylaxis (Beta=0.742; 95% CI 0.5471.007; *P*=0.055) was marginally significant.

**Conclusion:**

The predictors identified may support a closer postoperative follow-up and individualized planning for a safe discharge.

**Table:** 

Abbreviations, acronyms & symbols	
ARB	= Angiotensin II receptor blocker
ASA	= Acetylsalicylic acid
BMI	= Body mass index
CABG	= Coronary artery bypass grafting
CAD	= Coronary artery disease
CI	= Confidence interval
CKD	= Chronic kidney disease
COPD	= Chronic obstructive pulmonary disease
DM	= Diabetes mellitus
EuroSCORE	= European System for Cardiac Operative Risk Evaluation
HF	= Heart failure
IABP	= Intra-aortic balloon pump
MI	= Myocardial infarction
REVASC	= Registro de Revascularização Miocárdica
USA	= United States of America

## INTRODUCTION

Coronary artery bypass grafting (CABG) is the most common cardiovascular procedure in the world. In the United States of America (USA), 160,160 CABG were performed in 2017^[1]^. From August 2014 to December 2016, 54.1% of cardiac surgeries performed in Brazil were CABG^[2]^. In 2013, the mean rate of CABG in the countries of the Organization for Economic Co-operation and Development was 44 per 100,000 inhabitants^[3]^, whereas in Brazil, between 2000 and 2017, the gross rate of CABG financed by the Brazilian Unified Health System ranged between 15.1 and 18.3 per 100,000 inhabitants aged 20 years or more^[4]^.

After CABG, hospital readmission within 30 days ranges between 6.1% and 23.6% in countries such as the USA, Canada, Lebanon, and Turkey^[5-15]^. The causes of readmission include infection, pulmonary complications, myocardial infarction (MI), arrhythmias, and others^[6,9-11,15]^. Out of 288,059 patients submitted to CABG in the USA between 2013 and 2014, the average cost per readmission within 30 days was approximately US$13,500, with a mortality rate of 2.3%. Therefore, reducing readmission after CABG is a priority^[16]^.

Several studies have investigated the predictors of readmission within 30 days following CABG. Patient-related predictors include demographic data (*e.g*., age and gender), anthropometric data (*e.g*., weight), and clinical aspects (*e.g*., number of comorbidities). Surgery-related predictors include, *e.g*., surgical urgency, number of bypasses, and length of hospital stay^[5-15]^. However, to the best of our knowledge, in Brazil there are no robust studies that investigated predictors of readmission after CABG, in order to support multiprofessional interventions to minimize these rates.

This study aimed to identify predictive factors of hospital readmission within 30 days after CABG in a Brazilian center.

## METHODS

A secondary analysis of an electronic database was conducted in the largest private health center in Latin America, which has 1,084 beds and performs around 330 cardiac surgeries monthly. The hospital is located in the city of São Paulo (São Paulo, Brazil).

The sample was composed of data from the *Registro de Revascularização Miocárdica* (REVASC) electronic database, which contains 243 variables of 3,010 patients submitted to CABG by the 14 cardiac surgery teams of the institution, representing 69.6% of the total surgeries performed between July 2009 and July 2010.

The inclusion criteria were as follows: patients older than 18 years, submitted to isolated CABG, who had been discharged alive, and with a hospital length of stay of up to 10 days. Patients with unavailable data on risk factors and outcomes at the REVASC database were excluded.

The antecedent variables were selected from those available in the REVASC database and based on the results of 10 studies that investigated the predictors of hospital readmission within 30 days following CABG through multivariate analyses, published from 2013 to 2018^[5,7-15]^.

### Patient-Related Variables

Patient-related variables were gender (male/female), age (in years), ethnicity as stated by the patient himself (Caucasian/ African-American/Mixed [African-American and Caucasian]/ Asian/Other), anthropometric characteristics (body mass index [BMI] [Kg/m^2^]), use of medicines (oral anticoagulants, acetylsalicylic acid [ASA], ticlopidine, clopidogrel, antiplatelet agents, beta-blockers, cardiac glycosides, oral nitrates, intravenous nitrate, calcium channel blocker, angiotensinconverting enzyme inhibitors, angiotensin II receptor blocker [ARB], intravenous inotrope and/or statins), life habits - smoking (never smoked, past smoker [> 1 month], smoker [< 1 month]) -, family history of coronary artery disease (CAD) - angina, MI, or CAD when less than 55 years old in first-degree relatives -, diabetes mellitus (DM), DM treatment (none, diet, oral medications, insulin), dyslipidemia, chronic kidney disease (CKD), dialysis, hypertension, previous stroke, chronic obstructive pulmonary disease (COPD), peripheral artery disease, cerebrovascular disease, previous angioplasty/stent, previous MI, time since previous MI (< 6 hours, 6-24 hours, 1-7 days, 8-21 days, > 21 days), heart failure (HF), angina, angina classification (stable/ unstable), arrhythmia, cardiac catheterization, vessel disease (> 70%) (none/one/two/three), left coronary trunk injury (> 50%), injury in the proximal anterior descending coronary artery > 70%, mortality as estimated by the European System for Cardiac Operative Risk Evaluation (EuroSCORE)^[17]^, and use of intra-aortic balloon pump (IABP).

### Surgery-Related Variables

Surgery-related variables were use of preoperative antibiotic prophylaxis, surgery indication (elective/urgency/emergency), use of intraoperative antibiotic prophylaxis, number of anastomoses with venous conduits, number of anastomoses with arterial conduits (use of the internal thoracic artery [none/right/left/both]), number of anastomoses with distal arterial conduits, use of radial artery, use of the gastroepiploic artery, coronary endarterectomy (none/right coronary/anterior descendent/diagonal/circumflex/ marginal), extracorporeal circulatory support, degree of hypothermia (28 ºC/31 ºC/34 ºC/normothermia), duration of aortic cross-clamping, and duration of perfusion.

Postoperative aspects were blood product transfusion, need for orotracheal reintubation during hospital stay, antibiotic suspension within 48 hours of surgery, use of ASA, intravenous insulin in the first 24 postoperative hours, prophylaxis for deep venous thrombosis, thrombocytopenia (until discharge), serum creatinine, and length of hospital stay.

The outcome variable was 30-day all-cause hospital readmission after isolated CABG. The time between surgery and readmission was calculated as the date of readmission minus the date of surgery. When more than one readmission occurred in 30 days, the first documented readmission was considered.

The variables were described according to mean, standard deviation, quartiles, and minimum and maximum values for quantitative variables. As to qualitative variables, absolute and relative frequencies were used. The confidence intervals were calculated when appropriate.

The relationship between the outcome and the clinical and demographic variables was investigated by Student’s *t*-test for quantitative variables or by Chi-square (or Fisher’s exact) test for the qualitative variables. The relationship of the outcome with the variables with a *P*-value ≤ 0.2 in the univariate tests was investigated by a multiple logistic regression model. A significance of 0.05 was considered and, for the analyses, IBM SPSS Statistics for Windows, Version 25.0, Armonk, NY: IBM Corp. and R software were used.

This research project was approved by the Research Ethics Committee of the University (Protocol No. 2.795.813) and of the hospital where the study was conducted (Protocol No. 2.933.217). Confidentiality and anonymity of patient data were maintained.

## RESULTS

Among the 3,010 patients at REVASC database, 2,272 patients were older than 18 years, had undergone isolated CABG, and were discharged alive within 10 days after surgery. Of these patients, the most prevalent were male, Caucasian, with a mean age of 62.2±9.77 years, and BMI 27.39±4.23 (Table 1). The most common comorbidities in our sample were hypertension, dyslipidemia, and angina. Most patients used ASA and oral nitrates (Table 1).

**Table 1:** Main demographic and clinical characteristics (n=2272).

		n	%
Male gender		1616	71.13
Ethnicity	Caucasian	1926	84.77
	African-American	86	3.79
	Mixed (Caucasian and African-American)	228	10.04
	Asian	32	1.41
Comorbidities	Family history of coronary artery disease	703	30.94
	Diabetes	809	35.61
Diabetes mellitus treatment	None	52	2.29
	Diet	21	0.92
	Oral	489	21.52
	Insulin	247	10.87
Dyslipidemia		1011	44.50
Chronic kidney disease		98	4.31
Hemodialysis		20	0.88
Hypertension		1870	82.31
Stroke		104	4.58
Chronic obstructive pulmonary disease		137	6.03
Peripheral arterial disease		89	3.92
Cerebrovascular disease		35	1.54
Heart failure		39	1.72
Angina		1717	75.57
Angina classification	Stable	1323	58.23
	Unstable	394	17.34
Angioplasty/previous stent		217	9.55
Previous myocardial infarction		1060	46.65
Time since previous myocardial infarction	< 6 hours	22	0.97
	6-24 hours	3	0.13
	1-7 days	27	1.19
	8-21 days	128	5.63
	> 21 days	880	38.73
	Arrhythmia	100	4.40
Smoking	Never smoked	1008	44.37
	Past smoker (> 1 month)	901	39.66
	Smoker (> 1 month)	363	15.98
Medication	Oral anticoagulation	12	0.53
	Acetylsalicylic acid	1592	70.07
	Ticlopidine	25	1.10
	Clopidogrel	249	10.96
	Antiplatelet agents	124	5.46
	Beta-blockers	1472	64.79
	Cardiac glycosides	32	1.41
	Oral nitrates	1417	62.37
	Intravenous nitrates	71	3.13
	Calcium channel blocker	417	18.35
	Angiotensin-converting enzyme inhibitors	1088	47.89
	Angiotensin II receptor blocker	394	17.34
	Intravenous inotropic agents	2	0.09
	Statin	1537	67.65

A total of 195 (8.6%) patients were readmitted within 30 days of CABG, of which 123 (63.1%) were readmitted for non-cardiovascular causes and 36.9% for cardiovascular causes. The main causes of readmission were surgical wound infection (20.51%), HF (13.85%), and pneumonia (10.26%) (Table 2).

**Table 2:** Causes of readmission within 30 days after coronary artery bypass grafting (n=195).

Type of Complication	n	%
Cardiovascular	72	36.92
Hypertensive peak	1	0.51
Angina	6	3.08
Stroke	6	3.08
Pulmonary thromboembolism	6	3.08
Arrhythmia	7	3.59
Myocardial infarction	7	3.59
Others	12	6.15
Heart failure	27	13.85
Non-cardiovascular	123	63.08
Deep wound infection not requiring reoperation	9	4.62
Deep wound infection requiring reoperation	15	7.69
Pneumonia	20	10.26
Others	38	19.49
Superficial wound infection	40	20.51

In the univariate analysis, the characteristics that were associated with readmission with a *P*-value ≤ 0.20 were age (*P*=160), ethnicity (*P*=0,016), DM treatment (*P*=0.196), CKD (*P*=0.002), dialysis (*P*=0.041), hypertension (*P*=0.062), COPD (*P*=0.001), previous angioplasty/stent (*P*=0.171), time since previous MI (*P*=0.124), use of ASA (*P*=0.029), use of antiplatelet agents (*P*=0.126), use of ARB (*P*=0.069), mortality expected by EuroSCORE (*P*=0.015), preoperative prophylaxis (*P*=0.052), cardiac catheterization (*P*=0.098), duration of aortic cross-clamping (*P*=0.193), need for orotracheal reintubation (*P*=0.094), intensive care unit readmission (*P*=0.156), and postoperative transfusion of blood products (*P*=0.004).

In the logistic regression, the variables that remained significantly related to readmission were mixed ethnicity, African-American ethnicity, CKD, COPD, use of ASA, and postoperative transfusion of blood products. Preoperative antibiotic prophylaxis was marginally significant (Table 3).

**Table 3:** Logistic regression for readmission within 30 days after coronary artery bypass grafting (n=2272).

Variable	*P*-value	Beta	95% CI	
			Inferior	Superior
African-American/Caucasian	0.049	0.136	0.019	0.988
Mixed/Caucasian	0.030	1.613	1.047	2.458
Chronic kidney disease	0.005	2.214	1.269	3.865
Chronic obstructive pulmonary disease	0.003	2.095	1.284	3.419
Acetylsalicylic acid	0.050	1.418	1.000	2.011
Transfusion of blood products	0.011	1.515	1.101	2.086
Preoperative antibiotic prophylaxis	0.055	0.742	0.547	1.007

CI=confidence interval

## DISCUSSION

This study identified predictors of hospital readmission within 30 days of isolated CABG in a large cohort of 2,272 Brazilian patients. To the best of our knowledge, this is the first study that identified these factors in a large sample in Brazil. Although our results are limited by the inclusion of data from patients submitted to CABG in a single center, we believe the results can be generalizable, given that the demographic and clinical characteristics of our sample, as well as the 8.6% prevalence of readmission, is similar to that found in previous studies^[1,5-18]^.

Although the relationship between preoperative antibiotic prophylaxis and readmission was marginally significant, this relationship is clinically significant, considering that surgical wound infection was the main cause for readmission. In our sample, all patients underwent pre- or intraoperative antibiotic prophylaxis. However, only 60% of the patients received the antibiotic one hour before the incision and only 87.2% had the antibiotic repeated after four hours (data not shown), as indicated by the Centers for Disease Control and Prevention^[16]^ for prevention of surgical wound infection. Similar to our results, the most recent study based on the New York State Registry found that the main causes of readmission after CABG were infection (16.9%) and HF (12.8%)^[19]^. Other studies support surgical wound infection, HF, and pneumonia as frequent causes of readmission^[6,9,11,15]^.

Previous studies found a 16-74% higher chance of readmission in African-American patients^[8,9,14]^. In our sample, although mixed ethnicity was associated with a 61% higher chance of readmission, the African-American ethnicity was associated with an 86% lower chance of readmission, which can be explained by the inexpressive representativeness of patients who self-declared as African-American in our sample (3.8%), with only one patient being readmitted after the surgery.

In our sample, CKD increased the chance of readmission up to 2.21 times. Previous studies found that CKD^[15,17]^ and creatinine levels predict early readmission after CABG in a severitydependent manner: the increased chances of readmission ranged between 27% and twofold, depending on the creatinine level and need for dialysis^[5]^.

Similarly, the literature shows that not only COPD, but its severity, was an important determinant of increased chance of readmission after CABG^[5,12,15]^, ranging from 10% in mild COPD to 58% in severe COPD. In our study, having COPD increased the chance of readmission by more than twofold.

Although COPD compensation measures are wellestablished during preoperative clinical assessment to determine the indication of CABG^[19]^ and even though it is not possible to fully reverse airflow limitation in this kind of patients, rigorous preoperative screening using the results of six-minute walking test, pulmonary and cardiac rehabilitation, and interventions for adherence to inhaled medication can assist on reducing morbimortality in these patients. Such interventions require further studies with COPD patients^[5,20,21]^.

Previous meta-analyses including 2,399 patients show that the preoperative use of ASA is associated with a reduction in the risk of postoperative MI, without reducing mortality, but it also increases the risk of chest bleeding/drainage, the need for packed red blood cell transfusion, and bleeding-related reoperation after CABG^[22]^. Therefore, another variable that should be readily known upon admission is the use of ASA, which was associated with a 41% greater chance of readmission in our sample.

A previous study found that the need for transfusion of blood products by 25.1% of patients undergoing CABG was associated with a 76% greater chance of hospital readmission^[12]^. In our study, over 59% received blood components, which impacted on a 1.5-fold greater chance of readmission for these patients. In fact, patients with severe bleeding who are transfused two units of packed red blood cell after CABG have increased risk of adverse events that increase patient severity, such as acute kidney injury and surgical wound infection, prolonged use of inotropic drugs, and IABP^[(23,24].^

The predictors ethnicity, CKD, COPD, and use of ASA are variables easily obtained through patient reporting, which enacts prediction of the risk as soon as the patient is admitted, as well as the planning of postoperative follow-up. Transfusion of blood components complements the group of predictors that should be postoperatively evaluated, thereby signaling the need for closer monitoring.

By considering the predictors found in this study, cardiac programs to decrease the risk of readmission after CABG must be implemented, including several professionals, such as nurses, psychologists, and physiotherapists. These programs must be performed in all institutions to evaluate the patients before the surgery and prepare them and their families after the surgery to change modifiable risk factors and improve medication adherence and cardiac rehabilitation.

### Limitations

The main limitation of the study is related to the retrospective analysis of the database regarding to the full data collection and completeness of the outcomes.

## CONCLUSION

The predictors associated with a greater chance of hospital readmission within 30 days after isolated CABG were mixed ethnicity, use of ASA, transfusion of blood components, CKD, and COPD. The African-American ethnicity was associated with a lower chance of readmission. The association between preoperative antibiotic prophylaxis and a lower chance of readmission was marginally significant. Our data indicate that these patients require closer postoperative follow-up.

**Table:** 

**Authors' roles & responsibilities**	
CRNC	Substantial contributions to the conception of the work; analysis and interpretation of data for the work; drafting the work; agreement to be accountable for all aspects of the work in ensuring that questions related to the accuracy or integrity of any part of the work are appropriately investigated and resolved; final approval of the version to be published
VBS	Interpretation of data for the work; revising the work critically for important intellectual content; final approval of the version to be published
JLL	Interpretation of data for the work; revising the work critically for important intellectual content; final approval of the version to be published
CTL	Substantial contributions to the conception of the work; analysis and interpretation of data for the work; revising the work critically for important intellectual content; agreement to be accountable for all aspects of the work in ensuring that questions related to the accuracy or integrity of any part of the work are appropriately investigated and resolved; final approval of the version to be published
